# Chitosan and Cellulose-Based Hydrogels for Wound Management

**DOI:** 10.3390/ijms21249656

**Published:** 2020-12-18

**Authors:** Sibusiso Alven, Blessing Atim Aderibigbe

**Affiliations:** Department of Chemistry, University of Fort Hare, Alice Campus, Eastern Cape 5700, South Africa; 201214199@ufh.ac.za

**Keywords:** chitosan, cellulose, hydrogel, wound dressings, burn wounds, diabetic wounds, chronic wounds, acute wounds

## Abstract

Wound management remains a challenge worldwide, although there are several developed wound dressing materials for the management of acute and chronic wounds. The wound dressings that are currently used include hydrogels, films, wafers, nanofibers, foams, topical formulations, transdermal patches, sponges, and bandages. Hydrogels exhibit unique features which make them suitable wound dressings such as providing a moist environment for wound healing, exhibiting high moisture content, or creating a barrier against bacterial infections, and are suitable for the management of exuding and granulating wounds. Biopolymers have been utilized for their development due to their non-toxic, biodegradable, and biocompatible properties. Hydrogels have been prepared from biopolymers such as cellulose and chitosan by crosslinking with selected synthetic polymers resulting in improved mechanical, biological, and physicochemical properties. They were useful by accelerating wound re-epithelialization and also mimic skin structure, inducing skin regeneration. Loading antibacterial agents into them prevented bacterial invasion of wounds. This review article is focused on hydrogels formulated from two biopolymers—chitosan and cellulose—for improved wound management.

## 1. Introduction

Wounds are injuries on the skin [1]. They are classified as acute and chronic wounds. An acute wound is an injury to the skin that is sudden. It can heal within the time frame of 2–3 months, depending on its depth and size in the skin epidermis or dermis layers [2]. Chronic wounds are life-threatening because they fail to heal in a timely manner and examples of chronic wounds are burns, decubitus ulcers, infections, leg ulcers, etc. [1]. Wound management is expensive and there is an urgent need to design wound dressings that are affordable for the growing population. The United States of America spends about USD 20 billion annually for the management of chronic wounds, while the United Kingdom spent approximately GBP 184 million in 2012 for the management of chronic wounds [3,4,5]. Some of the currently used wound dressing materials suffer from several limitations such as poor antimicrobial effects, weak mechanical performance, and inability to provide moisture for acceleration of the wound healing process [1]. There is a pressing need for researchers to develop more advanced wound dressings that are cost-effective.

Wound dressings are mostly developed from biopolymers and synthetic polymers. The natural polymers that are commonly utilized include chitosan, cellulose, fibrin, elastin, hyaluronic acid, dextran, elastin, alginate, collagen, and gelatin [6]. These polymers possess interesting properties suitable for wound management such as good biocompatibility, non-toxicity, biodegradability, readily availability, and non-immunogenicity [7]. These polymers are usually cross-linked with synthetic polymers because of their poor mechanical properties. The synthetic polymers include poly(vinyl pyrrolidone) (PVP), poly(ethylene oxide)(PEO)/poly(ethylene glycol) (PEG), Poly(hydroxyethyl methacrylate) (PHEMA), poly(vinyl alcohol) (PVA), polyurethanes (PUs) and polyesters such as (polyglycolic acid (PGA), poly(lactic-co-glycolic acid) (PLGA) and polylactide (PLA)) [6,8]. In the design of an ideal wound dressing, factors that are usually considered are their ability to stop bleeding, prevention of microbial infections, good absorption of wound exudates, easy sterilization, their ability to promote wound debridement, good gas permeability, easy to use, biodegradability, and non-toxicity [9].

**Figure 1:**
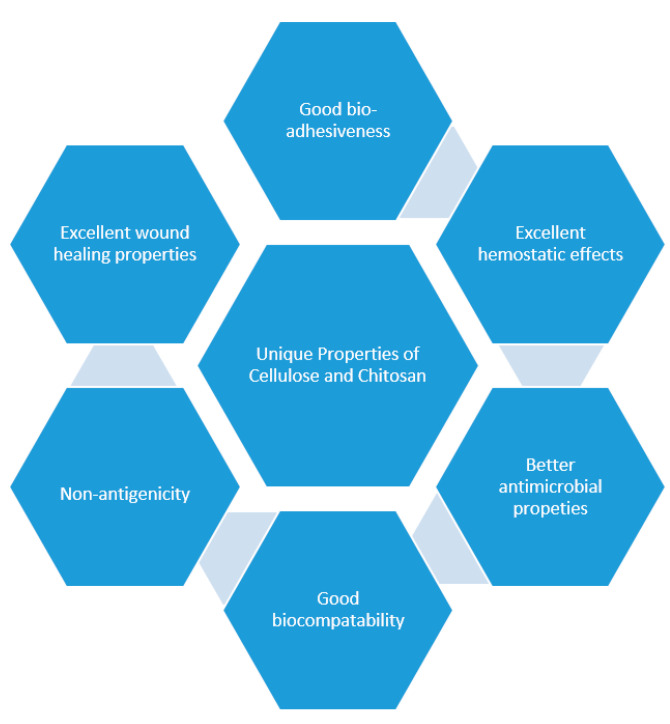
Properties of chitosan and cellulose.

Wound dressing materials are formulated in various forms such as hydrogels [10,11,12], films [13,14,15,16,17], nanofibers [18,19], foams, topical formulations, wafers [20], transdermal patches [21,22,23], sponges [24,25,26], and bandages [27,28,29]. The limitations that are associated with some of the currently employed wound dressings include poor gaseous exchange between the wound and the surroundings, difficulty in the removal of the wound dressing, inability to protect the wound against microbial infection, poor mechanical properties, lack of sterility, induction of allergic reactions, poor absorption of wound exudates, and their inability to sustain a moist environment for accelerated wound healing [30]. Most of the aforementioned features that are lacking in some of the currently designed wound dressings are exhibited by hydrogels developed from biopolymers such as chitosan and cellulose. There are various interesting unique properties of chitosan and cellulose that are attractive for the development of wound dressings. The properties include excellent hemostatic capability and antibacterial effects, availability of various types of functional groups that can be used for their modification, bio-adhesiveness, highly biocompatible, and outstanding wound healing characteristics (Figure 1) [6]. Some of the biopolymers are not presenting these properties. Combining the aforementioned features with the properties of hydrogels can result in an effective wound care. This review article is focused on the in vitro and in vivo therapeutic outcomes and properties of hydrogels that were recently developed (2019–2020) from chitosan and its derivatives, and cellulose and its derivatives for wound management.

## 2. Phases of Wound Healing

**Figure 2:**
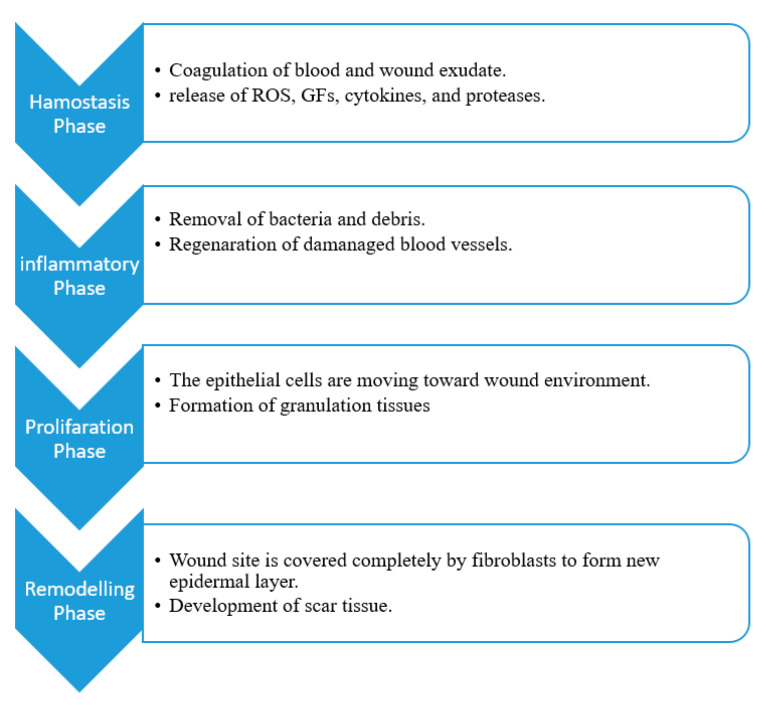
Summarized sequential phases of the wound healing process.

The wound healing process is a complex mechanism that leads to the repair of injured skin tissue. The phases of wound healing determine the type of wound dressing to be used for the proper management of the wound. The wound healing process is composed of four sequential phases that can overlap: hemostasis, inflammation, proliferation, and remodeling phase (Figure 2) [31]. The hemostasis phase takes place immediately after an injury, and this phase occurs rapidly. The blood vessel becomes narrow, restricting the blood flow. There is a platelet accumulation forming a clot that seals the ruptured wall of the blood vessel, thereby terminating the bleeding [32]. The inflammatory phase involves the cleansing of debris and bacteria removal by neutrophils, reactive oxygen species, and proteases which are released by phagocytic cells to provide a suitable environment for the healing process [33]. The hemostasis and inflammatory phase concurrently take place for 3 days [34]. The injured blood vessels produce transudate resulting in swelling. Inflammation is useful at this phase to prevent infection and control bleeding. However, prolonged inflammation can be problematic.

The proliferative phase involves the formation of connective tissue such as blood vessels, granulation tissues in the injury location to replace dead cells [35]. Extracellular matrix, including proteoglycans, elastin, hyaluronic acid, and collagen, produces a granulation tissue for the replacement of clot original formation. There are several types of cytokines and growth factors that participate in this phase, such as the transforming growth factor-β family (TGF-β, including TGF-β3, TGF-β1, and TGF-β2), angiogenesis factors, and interleukin (IL) family [35,36]. This phase normally takes place for days or weeks. The final step of the healing process of the wound is a remodeling phase. The collagen is remodeled by crosslinking to reduce the thickness of the scar with complete wound closure. Apoptosis occurs at this phase in which cells which were involved in the repair of the wound that are no longer useful are removed. This phase is very fragile and the inability of a wound to progress to this stage, results in a wound becoming chronic [37]. This significant phase can remain for months or even years [38,39].

## 3. Properties of Biopolymers

Biopolymers are derived from natural sources such as plant or animal and microbial sources [40]. They are biodegradable and it influences their physicochemical properties and their behavior (such as interaction with surrounding tissues, drug-releasing patterns, etc.) in the physiological environment [41]. The risks such as the transmission of infectious diseases from polymeric materials of natural origin can limit the use of biopolymer-based wound dressings in the wider applications of wound care due to their allogenic or xenogenic nature [42]. Other shortcomings of biopolymer-based systems include their poor stability and weak mechanical performance, which limits their biomedical applications [43]. Excitingly, there are numerous chemical modifications that have been advanced to overcome these shortcomings [44,45].

**Figure 3:**
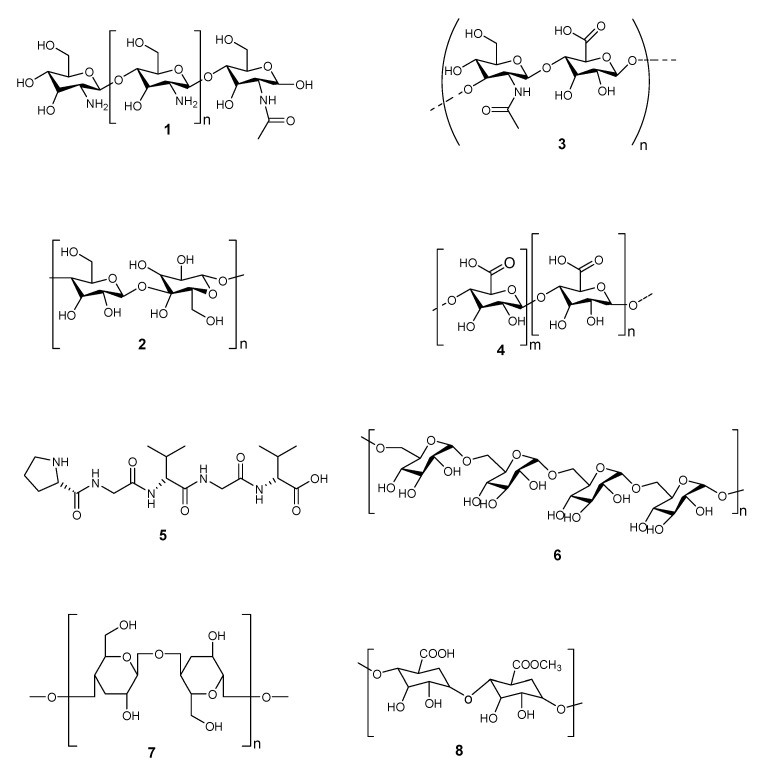
Molecular structures of biopolymers: chitosan **1**, cellulose **2**, hyaluronic acid **3**, alginate **4**, Elastin **5**, dextran **6**, fibrin **7**, pectin **8**.

Some examples of biopolymers that are usually utilized for the preparation of wound dressings include chitosan **1**, cellulose **2**, hyaluronic acid **3**, alginate **4**, Elastin **5**, dextran **6**, fibrin **7**, pectin **8**, and collagen (Figure 3) [6]. Chitosan is a linear copolymer isolated from chitin, which is the key constituent of the exoskeletons of crustaceans such as crab and shrimp. This biopolymer and its derivatives are well-acknowledged for their heterogenic functionalities such as their non-toxicity, inertness, non-antigenicity, bioadhesiveness, biocompatibility, biodegradability, hemostatic effects, antimicrobial properties, and wound healing characteristics [46,47]. Additionally, chitosan is greatly versatile and possesses the ability to make a diversity of functionalized derivatives through the chemical modification of hydroxyl and amino groups. These derivatives include N,O-(carboxymethyl), N-carboxymethyl, N-succinyl, N-acyl, N-carboxybutyl, N-carboxyethyl, 5-methylpyrrolidinone, N-N-dicarboxymethyl, O-succinyl, and O-carboxymethyl chitosan derivatives etc.

On the other hand, cellulose is the major structural constituent of the cell walls of plants and is well-known as the most abundant biological polymer in the universe. This biopolymer is readily accessible and affordable. It is a linear organic polymer composed of β-1,4 combined D-glucose units which are linked to produce cellobiose repeating parts [48]. Cellulose and its derivatives display good biocompatibility [49]. Furthermore, the resorption of cellulose in cells does not happen since cells are unable to produce enzymes, cellulases [50]. The wound healing efficacy of cellulose results from its capability to accelerate the wound healing process through the maintenance and release of several growth factors at the injury site such as basic fibroblast growth factor, phosphodiesterase growth factor, and epidermal growth factor. These growth factors promote the migration and proliferation of dermal fibroblasts and inhibit the proliferation of bacteria in the wound [51].

## 4. Preparation Techniques and Properties of Hydrogels

There are four techniques that are adopted for the preparation of hydrogels: chemical crosslinking, physical crosslinking, radiation cross-linking, and grafting polymerization. These techniques can enhance their viscoelasticity and mechanical properties for their application in pharmaceutical and biomedical fields [52]. Chemical cross-linking can be defined as the incorporation of monomers on the polymers or link of two polymer chains using cross-linking agents. The cross-linking of synthetic and biopolymers can be performed through the reaction of their functionalities such as carboxylic, amine, and alcohol groups with the help of cross-linkers such as aldehydes (e.g., adipic acid dihydrazide and glutaraldehyde). The major methods under chemical cross-linking include grafting, crosslinker, and radiation in aqueous and/or liquid state which are employed to formulate hydrogels from a range of biopolymers [52].

Physical crosslinking has been of great interest in gel development because of the ease of formulation and the benefit of not utilizing cross-linking agents. The careful selection of pH, concentration, and hydrocolloid type can result in the development of a wide range of gel textures and is presently an area of great attention. The methods used to develop physically cross-linked hydrogels include cooling/heating a polymer solution, ionic interaction, complex coacervation, hydrogen-bonding and maturation freeze–thawing [52]. Radiation crosslinking is a broadly employed technique that does not require the use of chemical additives and therefore maintains the biocompatibility of the natural polymer. In addition, sterilization and modification can be performed in a single step, and hence it is an affordable process for natural polymer modification. This technique mainly depends on generating free radicals followed by exposure to a high energy source such as electron beam, X-ray or gamma ray. The direct or indirect action of radiation will rely on the polymer environment (i.e., solid-state, dilute solution, concentration solution). The major methods under radiation cross-linking include aqueous state radiation, radiation in a paste, solid-state radiation [53]. Graft polymerization is a process whereby monomers are bonded covalently and polymerized as side chains into the main polymer backbone. Grafting is an interesting approach that imparts a diversity of functionalities to a polymer. Graft polymers are also known as graft copolymers and comprise of at least two different types of monomer parts, such as the grafted side chains that are structurally different from the main chain. The monomer to be grafted may be of one or more than one kind; thus, the graft chains in the grafted copolymer may be homopolymers or copolymers [54].

**Figure 4:**
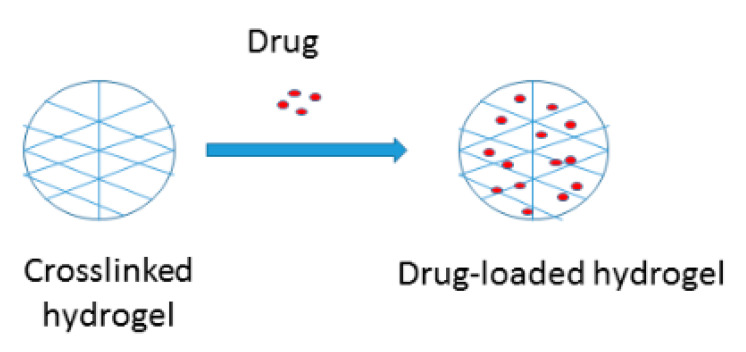
Schematic diagram of hydrogels.

Hydrogels can be defined as hydrophilic wound dressing scaffolds with three-dimensional networks (Figure 4) that can be designed from biopolymers and synthetic polymers. These wound dressings possess the capability to absorb a huge volume of water and biological fluids [55]. Hydrogels have been extensively utilized for applications in wound dressings because of their good porosity, ability to load and release various drugs, to offer debriding and desloughing capacity on fibrotic and necrotic tissue, to provide a moist environment, good flexibility, biocompatibility, and high water content [56,57]. The advantages of hydrogels also include soothing outcomes that enhances patient compliance, effective in softening necrotic tissue on the wound surface, hydrate wound surfaces, and they can be used or removed without interfering with the wound bed and they are non-adherent.

Although there are several interesting properties of hydrogels in the field of wound management, they also suffer from some limitations such as dehydration if they are not covered, result in skin maceration, they need a secondary dressing, it is not easy to secure them, and they possess poor mechanical stability at swollen state [58]. Other disadvantages of hydrogels are their ineffectiveness in wounds with excessive wound exudates, which may involve secondary wound dressings and they are not suitable scaffolds for moderate-to-extreme exudative wounds [58].

### 4.1. Chitosan-Based Hydrogels

**Figure 5:**
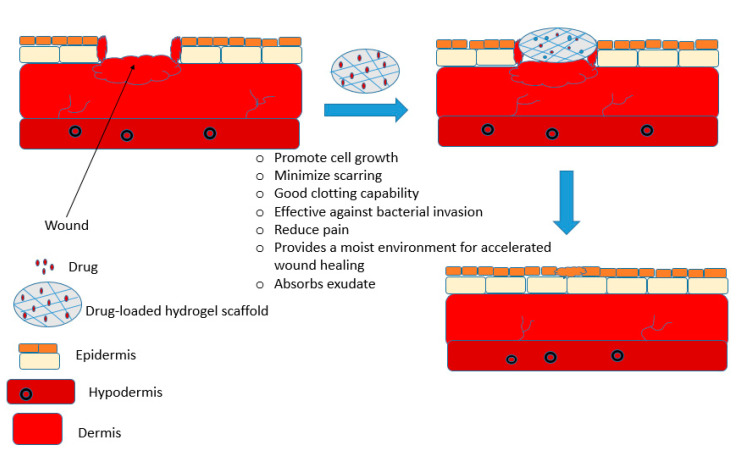
A schematic diagram illustrating the efficacy of chitosan-based hydrogel as an effective wound dressing.

There are several reports of chitosan-based hydrogels for the management of wounds with good healing outcomes (Figure 5) (Table 1). Rasool and co-workers designed stimuli-responsive chitosan-based hydrogels by blending poly (N-vinyl-2-pyrrolidone) (PVP) and chitosan using solution casting technique for potential application in wound healing [59]. The hydrogels were thermally stable when compared to the free polymers. The hydrogels were biodegradable and pH-sensitive, with a maximum swelling degree of 10,220%. Their swelling capability increased with the increase in the pH and a decrease in swelling at neutral pH. The antimicrobial activity of the hydrogel samples was investigated using the disc diffusion method and all the chitosan-based hydrogels possessed antibacterial efficacy against Gram-negative *E. coli* strains which is attributed to the interaction of chitosan with the plasma membrane of the bacteria. Chitosan molecules have positively charged NH_3_^+^ groups and *E. coli* outer covering is composed of lipopolysaccharides that influence the significant negative charge of the bacterial surface. Furthermore, chitosan binds with bacterial DNA, thereby inhibiting transcription and translation. The in vitro drug release profile of the hydrogels loaded with antibiotics, silver sulfadiazine used in burn wound was sustained and controlled with over 91.2% of the drug released in 1 h. The results demonstrated that the chitosan-based hydrogels are potential scaffolds for burn wound management [59].

Xue and co-workers prepared chitosan–Matrigel–polyacrylamide hydrogels for wound healing and skin regeneration. These hydrogels showed good porous morphology with pore sizes ranging between 10 and 30 μm, which can significantly maintain a moist environment useful for cell migration, adhesion, and viability. These hydrogels showed good mechanical properties such as high fracture strain (1210%), compression, tensile strength (118.3 kPa), and resilience. The adhesive strength of the hydrogels on porcine skin was 1.3 kPa indicating that they can be easily removed from the wound surface without causing further trauma to the wound surface. The hydrogels swelling capability reached an equilibrium state of approximately 1200% after two days. The antibacterial activity of the hydrogels showed significantly better growth inhibition against *S. aureus*, *E. coli,* and *S. epidermidis* strains when compared to the control, kanamycin. The hydrogels reduced the amount of blood loss significantly in bleeding mouse liver. The wound healing capability of the hydrogels in vivo showed good wound contraction on the 5th day when compared to three control groups [60].

Khorasan and co-workers formulated chitosan/polyvinyl (alcohol)/zinc oxide hydrogels using the freeze–thaw method. The hydrogels were porous, with a mean pore size of 13.7 ± 5.9 μm. Their water absorption ranged between 680% and 850%. The mechanical properties of chitosan hydrogels showed decreased elastic modulus and tensile strength with increased elongation at the breakpoint. Their antibacterial efficacy result displayed the highest inhibition zone in *S. aureus* when compared to *E. coli* strain. The in vitro wound healing experiments of the prepared hydrogels displayed no toxicity and accelerated wound healing phase was significant [61]. Masood et al. designed chitosan-polyethylene glycol (PEG) hydrogel entrapped with silver nanoparticles for diabetic wound management. Their average particle size was 99.1 ± 2.3 nm. The hydrogel showed significant high porosity of 72.2%, which contributed to the accelerated wound healing, indicating a good oxygen penetration and absorption of exudates. The high swelling capacity of the plain hydrogels was significant when compared to nanoparticle-loaded hydrogels [62].

The silver nanoparticle-loaded hydrogels demonstrated a water vapor transmission rate of 2104 g·m^−2^·24 h^−1^ and 1391.3 g·m^−2^·24 h^−1^ for the plain hydrogels indicating the suitability of nanoparticle-loaded hydrogels as a potent wound dressing for wound management. The in vitro release mechanism of nanoparticles from the chitosan-based hydrogels was slow and sustained at 37 °C. The antimicrobial activity evaluations demonstrated that chitosan-based hydrogels loaded with silver nanoparticle have good antimicrobial efficacy compared to the plain hydrogels and nanoparticles. The nanoparticle-loaded hydrogels exhibited higher zone inhibition of 21.5 ± 0.5, 15.5 ± 0.8, 21.8 ± 1.5, and 20.2 ± 1.0 mm against *S. aureus*, *B. subtilis*, *P. aeruginosa* and *E. coli*. The wound healing experiment exhibited that the loading of silver nanoparticles in chitosan-based hydrogels significantly improved the wound healing in diabetes-induced rabbits. The loaded hydrogels showed 47.7% wound contraction after 4 days compared to 12.6% in the negative control [62].

Wenbo et al. designed chitosan–heparin hydrogel for controlled release of SDF-1 α for endometrium wound injury healing in a mouse model. The results of Western blots assay, Masson trichrome staining, H&E staining, immunofluorescence staining and immunohistochemical staining demonstrated that the endogenous c-kit positive stem cells adhered to the wounded spot and stimulated the wound healing process. The in vitro drug release profile of DF-1α from the hydrogel was 5% in the 1st hour, and increased to 18 and 53% after 24 h and 102 h, respectively, indicating the slow and controlled release mechanism [63].

Zhang et al. prepared and evaluated carboxymethyl chitosan composite hydrogels. The results from the in vitro studies of the effects of composite hydrogels on the development of Human skin fibroblasts NS-FB and human hypertrophic scar fibroblasts (HS-FB) cells demonstrated the outstanding mechanical properties and good biological activity of chitosan-based hydrogels, and their potential application for the development of wound dressing. The highest swelling ratio of the hydrogel was over 581% with good water retention ability. The hemolysis rate of the hydrogel was 0.81–2.67% revealing good blood compatibility. The survival rate of NS-FB cells cultured by the hydrogel extract was greater than 93% showing good cytocompatibility and indicating their capability to induce cell proliferation. The survival rate of HS-FB cells was greater than 88% with no adverse side effects [64]. Chitosan-based hydrogels formulated by Zhang et al. showed modulating cationicity of chitosan with the capability to inhibit hypertrophic scar development during the wound healing process. The hydrogels were loaded with varying concentrations of genipin in the range of 2.5–15%. The hydrogel inhibited hypertrophic scar via suppression of the expression of a smooth muscle actin and induction of type I matrix metalloproteinases. The incorporation of 2% (*v*/*v*) Aloe vera gel further enhanced the inhibition of scar formation. The cationicity of the chitosan hydrogels is useful in the proliferation and differentiation of human skin fibroblasts, the formation of ECM and the production of growth factors [65].

Bagher et al. designed alginate/chitosan-based hydrogels loaded with hesperidin for wound healing in a rat model. These hydrogels displayed suitable porosity of 91.2% with interrelated pores, appropriate swelling capacity, and biodegradability confirmed by weight loss of over 80% after 2 weeks. The in vivo wound healing studies demonstrated that the formulated hydrogels had a better wound closure when compared to the gauze-treated wound, especially the hydrogels loaded with 10% of hesperidin [66]. Ehterami et al. reported similar findings as Baghe et al. for alginate/chitosan-based hydrogel loaded with Alpha-tocopherol for wound management in vivo [67]. The porosity of the hydrogel was 89.2% with interconnected pores. It was biodegradable with a weight loss of 80% in two weeks. The wound closure was accelerated when compared to the gauze-treated wound (the control). Neo-tissue and granulation tissue formation was visible in vivo when using the hydrogel on animal models [67].

He et al. prepared adhesive nanocomposite hydrogels via crosslinking of N-carboxyethyl chitosan and Pluronic F127 for wound healing. These hydrogels displayed remarkable photothermal antibacterial activity on *E. coli* and *S. aureus* infected wound in vivo [68]. These hydrogels displayed stable mechanical properties, suitable gelation time, good biodegradability, high water absorbency, and hemostatic properties. The hydrogels exhibited a pH-responsive drug release mechanism and good antimicrobial efficacy after they were loaded with antibiotic moxifloxacin. The hydrogel tissue adhesive property allowed these hydrogels to have an excellent hemostatic outcome in a mouse liver trauma model, mouse tale amputation model and mouse liver incision model. Furthermore, the in vivo assessment in a rat full-thickness skin wound-infected model showed that these formulations are excellent wound dressings for improved wound closure, angiogenesis, and collagen deposition [68]. Hamdi et al. formulated carotenoids enriched blue crab chitosan composite hydrogels. The release of the carotenoids was enhanced at pH 7.4 when compared to pH 4.0 and 2.0 showing that the hydrogels are pH-sensitive intelligent drug delivery systems. The in vivo wound healing assessments of the carotenoids enriched chitosan-based hydrogels in rat models demonstrated the acceleration of wound and complete healing. The hydrogels displayed low toxicity [69].

Du et al. prepared injectable chitosan-based hydrogels for application in wound healing. The hydrogels were active against *P. aeruginosa* and *S. aureus* and demonstrated killing efficiencies of 96.4% and 95.0%, respectively, at bacteria concentration of 108 CFU/mL. After two weeks, the wounds treated with the hydrogel displayed a 99.8% wound closure which was characterized by skin hair. The hemostatic activities of the hydrogels on a rat liver hemorrhaging model showed that the hydrogel adhered quickly to the tissue surrounding the bleeding site resulting in the formation of a barrier that prevents hemorrhaging. These results revealed that chitosan-based hydrogels are suitable for hemorrhagic healing and bacterially infected wound healing [70]. Zhang et al. formulated and evaluated novel chitosan–PVA–lignin composite hydrogels for wound management [71]. The hydrogels displayed good antibacterial activity and biocompatibility. The addition of lignin in these hydrogels effectively enhanced a wound moist environment for accelerated wound healing. The tensile strength was over 46.87 MPa. The hydrogels exhibited accelerated wound closure of 41% and 87% on the 5th and 10th day, respectively [71]. Pawar et al. designed chitosan-based hydrogels covalently incorporated with cefuroxime via ester bond for the treatment of infected wounds. The in vitro release profile of cefuroxime from the hydrogels was higher in alkaline medium of pH 10 and phosphate buffer of pH 7.4 with esterase enzyme when compared to the phosphate buffer. The drug release was via chemical and enzymatic hydrolysis. The hydrogel exhibited good biocompatibility on MG63 osteosarcoma and L929 fibroblast cell lines. The in vitro antibacterial activity against *S. aureus* revealed a high inhibition zone revealing that these hydrogels can be used for the treatment of infected wounds [72].

Pham et al. synthesized thermal-responsive chitosan-based hydrogels co-loaded with gelatin and curcumin for wound healing application. The in vitro drug release profile was sustained and controlled for curcumin and gelatin from the hydrogels. The in vivo evaluation demonstrated that these hybrid hydrogel displayed a synergistic effect of curcumin and gelatin, which stimulated the regeneration of the structure and the barrier’s function of injured skin such as a wound [73]. Ferreira et al. prepared chitosan hydrogels loaded with 2 and 4% chlorhexidine. The formulation displayed 100% inhibition of *Staphylococcus aureus* growth. In vivo studies showed that on day 14, the animal models treated with the hydrogel containing 2% chlorhexidine displayed high contraction of the wound compared to the control groups [74]. Another study of hMSCs-seeded injectable chitosan hydrogel scaffolds conducted by Xu et al. demonstrated improved full-thickness cutaneous wound healing. The histological detection of these hydrogels further showed that the hydrogels encapsulated with hMSCs considerably accelerated wound closure, re-epithelialization, microcirculation, hair follicle regeneration, tissue remodeling, and inhibited over-inflammation in the surrounding and the central wounds [75]. Ragab et al. formulated soft hydrogels encapsulated with *P. granatum* peel extract that is based on chitosan for the treatment of chronic wound. These biopolymer hydrogels displayed tolerable cytotoxicity against human fibroblast cells. The in vivo wound healing study using a rat model showed good healing process [76].

Long et al. formulated 3D-printed chitosan–pectin hydrogels for drug delivery of lidocaine hydrochloride for wound treatment. The in vitro release profile at physiological conditions displayed burst release in 1 h, followed by sustained and controlled release over a period of 4 h. The burst release of lidocaine over 1 h from these hydrogels offer an effective pain relief [77]. Viezzer and co-workers formulated and evaluated chitosan-based polyurethane hydrogels loaded with transplant bone marrow mesenchymal cells for chronic wound treatment. These hydrogels demonstrated enhanced wound healing of ulcers in a diabetic rat model with a significant wound closure [78]. Moradi et al. synthesized and characterized chitosan-based hydrogels encapsulated with thymine oil cyclodextrin inclusion compound. The hydrogels displayed high elasticity and less stiffness. A significantly decreased number of bacteria was observed after inoculation with chitosan-based hydrogels loaded with thymine oil, indicating their potential use for the management of bacterially infected wounds [79]. The immersion of wounds in seawater containing salt and pathogenic bacteria, can result in serious infections. Wang et al. prepared composite hydrogel from a combination of hyaluronic acid and quaternized chitosan to accelerate healing of wounds immersed in seawater. The hydrogel inhibited bacterial growth. The in vivo study in a seawater-immersed wound defect model showed that the hydrogels decreased pro-inflammatory factors and promoted anti-inflammatory factors (TGF-β1) in the wound. The hydrogel promoted excellent re-epithelialization, increased the granulation tissue thickness and the density collagen deposition with good antibacterial activity [80].

Chen et al. developed citric-modified chitosan hydrogel loaded via a freezing and thawing treatment method and loaded with tetracycline hydrochloride. The hydrogel antimicrobial activity was significant against *E. coli* and *S. aureus*. The tensile strength and modulus of the hydrogel were 2.01 ± 0.01 MPa and 16.12 ± 0.11 MPa (3 wt%), respectively, when compared to the native skin with tensile strength of 3 ± 1.5 MPa and modulus in the range of 2.16 kPa–0.1 MPa. In vivo studies showed that granulation tissue was visible on the wounds covered with hydrogels after 12 days [81]. Huang et al. physically blended *Bletilla striata* and carboxymethyl chitosan with Carbomer 940 for good gel formation and improved water retention. The hydrogel exhibited good blood compatibility with 85% cell viability on M293T cells revealing the non-cytotoxicity. The full-thickness wound studies showed a 71.64 ± 6.64% and 83.80 ± 5.56% wound healing rates on day 7 and day 14, respectively. The connective tissues formed were dense with a well-developed sebaceous gland, granulation tissue and hair follicle [82]. Nguyen et al. reported hydrogel composed of chitosan, polyvinyl alcohol, and loaded with silver nanoparticles. The hydrogel excellent antibacterial activity was against *P. aeruginosa* and *S. aureus* [83]. Chen et al. developed carboxymethyl chitosan-based hydrogels loaded with melatonin. Evaluation in a full-thickness cutaneous wound model showed increased wound closure with enhanced proliferation of the granulation tissue, re-epithelialization, and rapid collagen deposition. The hydrogel-induced angiogenesis with the enhanced expression of vascular endothelial growth factor receptor protein and cyclooxygenase-2. The hydrogel was highly porous with interconnected interior structure and a pore size of 250 μm [84]. Nooshabadi et al. developed chitosan-based hydrogel loaded with exosomes. The hydrogel wound closure ability was 83.6% in full-thickness excisional wound model when compared to the sterile gauze that showed 51.5%. A high degree of re-epithelialization was observed with a reduction in wound size [85]. Ravishankar et al. crosslinked chitosan using alkali lignin. The electrostatic interaction between the phenoxide groups in lignin and the ammonium groups on the chitosan contributed to the ionotropic cross-linking. The gels were non-toxic to the *Mesenchymal* stem cells. The hydrogels supported good cell migration indicating their potential application for tissue regeneration [86]. Lin et al. prepared hydrogel from poly (vinyl alcohol) (PVA), dextran, and chitosan using glutaraldehyde as a cross-linker. Preparing the hydrogel from 6% PVA and 0.25% chitosan enhanced the antimicrobial capability. Combining the hydrogel with 4% dextran induced high cell proliferation. The thermostability, water retention capability, mechanical properties, and moisturizing ability of the hydrogel was influenced by the addition of chitosan [87]. Heimbuck et al. reported genipin-cross-linked chitosan hydrogels. The average water uptake was ∼230% with good bacterial activity hindering 70% *E. coli*. It was biocompatible on fibroblast and keratinocyte cells in vitro. It induced high immune response and cellular proliferation in pressure wounds model used [88]. Ternullo et al. incorporated liposomes into hydrogel for dermal delivery of curcumin. The system displayed sustained delivery of curcumin with increased retention at the skin. The hydrogel bioadhesiveness was significant. Incorporation of neutral deformable liposomes in the hydrogel promoted the high bioadhesive nature of the hydrogel. The positively charged deformable liposomes promoted the bioadhesiveness and the sustained delivery of curcumin in ex vivo full human skin [89]. Qu et al. prepared hydrogel from N-carboxyethyl chitosan (CEC) and oxidized hyaluronic acid-graft-aniline tetramer. The hydrogels exhibited high swelling capability, good biodegradation property, and free radical scavenging capacity. The loading of amoxicillin prevented wound infection. It significantly accelerated wound healing rate with high granulation tissue thickness and more angiogenesis in a full-thickness skin defect model [90]. Cardoso et al. loaded phenytoin, an antiepileptic drug used for the treatment of epilepsy into chitosan hydrogels for the treatment of diabetic and burn wounds. The release of phenytoin was controlled from the hydrogel with good adhesion to the skin. Phenytoin promoted the formation of collagen fibers and fibroblasts in the wound site in vivo. The results reveal that phenytoin can be repurposed for wound healing [91]. Zahid et al. prepared hydrogel from chitosan, polyvinyl alcohol and S-nitroso-*N*-acetyl-DL-penicillamine. It induced significant angiogenesis in chronic wounds with sustained production of NO. The production of NO promoted angiogenic activity and accelerated wound healing. It supported the proliferation of 3T3 and HaCaT cells in vitro [92].

Bano et al. reported chitosan–PVA soft membranes for the treatment of burn wounds. The hydrogel supported normal growth of epidermis and accelerated the formation of granule and fibrous connective tissues. Accelerated wound healing and re-epithelialization of burn wounds resulted in a decreased deposition of collagen thereby preventing severe scar formation [93]. Nešović et al. prepared chitosan-poly(vinyl alcohol) hydrogels via a freezing–thawing method and loaded it with silver nanoparticles. The hydrogels were non-cytotoxic with good antibacterial activity against *S. aureus* and *E. coli*. Their swelling capability was improved by increasing the content of chitosan and the addition of AgNPs. The release profile of the drug from the hydrogel was an initial burst release followed by a slow drug release from day 5 to day 28. The release profile was appropriate for the effective treatment of a bacterially infected wound followed by a slow drug release thereby providing a sterile wound environment for an extended period [94]. Du et al. reported chitosan-based hydrogel prepared from hydrocaffeic acid-modified chitosan with hydrophobically modified chitosan lactate for sutureless closure of surgical incisions. The hydrogel was effective against *S. aureus* and *P. aeruginosa* in vitro. It was non-cytotoxic on 3T3 fibroblast cells, biocompatible and biodegradable. It exhibited good In situ antibleeding efficacy in rat hemorrhaging liver and full-thickness wound models. It closed the wound in a sutureless way thereby promoting wound healing [95]. Mousavi et al. developed chitosan/gelatin hydrogels in the ratio of 1:5 and 1:1, respectively. The presence of collagen in the hydrogel reduced the swelling capability, biodegradation rate of the hydrogels and the mechanical strength when compared to the gelatin. Collagen loaded in the hydrogel promoted cell attachment [96].

Wang et al. reported hydrogel prepared from a combination of hyaluronic acid and quaternized chitosan to promote wound healing and prevent bacterial infection. The biocompatibility of the hydrogels in vitro on fibroblast L929 cell was significant. The hydrogels displayed high repair in a seawater-immersed wound defect model. A decreased expression of pro-inflammatory factors such as (TNF-α, IL-1β, and IL-6) and increased expression of TGF-β1, an anti-inflammatory factors was significant in the wound. The hydrogel was effective against *S. aureus* [97]. The wound healing was accelerated and characterized by excellent reepithelialization, high deposition of collagen, good thickness of the granulation tissue and low level of endotoxin. Martínez-Ibarra et al. prepared hydrogels from a combination of xyloglucan and chitosan. The pore sizes of the hydrogels were in the range of 32.8–101.6 μm. The hydrogels were biodegradable with a significant weight loss over a period of two weeks. Their antibacterial activity was influenced by the biopolymers used for their preparation [97]. Soriano-Ruiz et al. prepared chitosan hydrogel for epidermal regeneration. The porous microstructure of the scaffold enhanced adequate oxygen and nutrient diffusion to the wound. Furthermore, hMSCs encapsulation provided an appropriate microenvironment that supported their viability for 7 days. The viability of hMSCs was above 75% [98]. Patil et al. investigated the benefit of chitosan hydrogels with or without oxygen. Wound healing processes are either directly or indirectly dependent on oxygen such as inflammation, angiogenesis, phagocytosis, cell proliferation, collagen synthesis etc. In vivo studies on an acute porcine wound model of the hydrogel was compared with a control, Derma-Gel™ hydrogel dressings. The combination of the hydrogel with oxygen accelerated the wound closure when compared to the control. The combination of the hydrogel with oxygen enhanced the formation of new blood vessel formation with maturation of keratinocyte [99]. Li et al. prepared collagen/chitosan gel composite loaded with cell-penetrating peptide (Oligoarginine, R8). The hydrogel composite inhibited *Staphylococcus aureus* growth. In vivo studies on animal models showed a complete wound surface healing rate of 98% on day 14. The formulation promoted the enhanced formation of granulation tissue, increased deposition of collagen and angiogenesis was visible in the wound tissue [100].

Soares et al. developed chitosan-based hydrogel loaded with a mixture of flavonoids obtained from Passiflora edulis Sims leaves for the treatment of diabetic wounds. In vivo wound healing studies on male Wistar rats showed that the treatment of the wound with the hydrogel stimulated the antioxidant defense systems. The release of the flavonoid from the hydrogel was rapid. On the 14th day of treatment, a high oxidative stress was visible which is attributed to hypoxia caused at the wound site due to the presence of the film. The hydrogel promoted increased lipid peroxidation at the injured tissue [101]. Movaffagh et al. reported chitosan hydrogel loaded with honey. The hydrogels were effective against *Staphylococcus aureus*, *Bacillus cereus*, *Escherichia coli*, *Pseudomonas aeruginosa*, and *Candida albicans*. The combination of honey with chitosan accelerated the wound healing process and enhanced the antibacterial activity [102]. Djekic et al. prepared chitosan hydrogel by ionic gelation technique. It was loaded with ibuprofen and the in vitro release profile of the drug was sustained over a period of 12 h followed by a zero-order release mechanism. The hydrogel displayed good cohesiveness and adhesiveness [103]. Lim et al. studied the wound healing efficacy of thermosensitive hydroxybutyl chitosan hydrogel loaded with human platelet lysate. The release of human platelet-derived growth factor from the hydrogel was sustained. The hydrogel promoted wound healing in vivo with the formation of new collagen. The hydrogel promoted a high level of Human Umbilical Vein Endothelial Cells proliferation and tube formation in vitro [104]. Li et al. reported IGF-1C domain-modified chitosan hydrogel for skin regeneration. The hydrogel had excellent proangiogenic effects with an accelerated wound closure and elevated remodeling of the extracellular matrix. It accelerated the cutaneous wound healing via stimulating angiogenesis [105].

Yan et al. prepared chitosan-gentamicin conjugate hydrogel with good water solubility and excellent antimicrobial activity. The hydrogel was effective against *P. aeruginosa* and *S. aureus* with the diameter zone of inhibition of 20.3 ± 0.06 mm and 20.0 ± 1.0 mm, respectively. The cell viability studies showed that the hydrogel co-incubation with L929 cells did not induce toxic effect (at a concentration 200 µg/mL) with cell viability of 101.68%, 100.44%, 86.73%, respectively, on day 1, 2 and 3. The hemocompatibility of the hydrogel was superior when compared to the free drug. On day 14 and 21, the wound healing rate was 89.18% and 99.61%, respectively, in vivo on the animal model treated with the hydrogel. On day 14, complete epidermal regeneration was visible [106]. Cifuentes et al. reported the efficacy of chitosan hydrogels functionalized with either bemiparin or unfractionated heparin for diabetic wound healing. The hydrogels accelerated the inflammation process and enhanced the re-epithelialization. The thickness of the neodermal in the animal model after treatment with the hydrogel was similar to the non-diabetic animal models. The hydrogels healing effect was similar. However, treating the animal model with chitosan hydrogel loaded with bemiparin resulted in good quality of tissue in the neoformed dermal tissue when compared to the chitosan hydrogels functionalized with unfractionated heparin [107].

Zhang et al. developed a temperature-responsive hydroxybutyl chitosan-based hydrogel from chitosan and dopamine at different concentrations. The hydrogels were non-toxic to mouse fibroblast cells (L929). The hydrogel was effective against *S. aureus* with good blood clotting capability [108]. Kalantari et al. prepared polyvinyl alcohol/chitosan hydrogel loaded with cerium oxide nanoparticles. The hydrogel with 0.5% CeO_2_-NPs was effective against the growth of MRSA. It was non-toxic to human dermal fibroblasts healthy human dermal fibroblast with high viabilities of 90% over a period of 5 days [109]. Leonhardt et al. reported chitosan-loaded hydrogels that degrade at the site of the injury. The diameters of the hydrogel was 9.2 ± 3.7 nm resulting from an increase in the surface area of chitosan. The good hemostatic capability of the hydrogel revealed their promising application to control blood loss in skin injury [110]. Wang et al. developed chitosan/oxidized konjac glucomannan hydrogel loaded with silver nanoparticles for the treatment of irregular wounds. A comparative in vivo study of the hydrogel with a commercial hydrogel dressing (AquacelAg™) showed that the hydrogel displayed self-healing ability, good tissue adhesiveness, and antibacterial activity, making it a self-adapting wound dressing for the treatment of irregular wounds [111].

Yang et al. developed chitosan hydrogels loaded with LL-37 peptide for the treatment of pressure ulcer. The hydrogel at a concentration of 5 μg/mL was effective against *S. aureus* [112]. In vivo studies on animal models revealed high epithelial thickness and newly-formed capillary at day 15 and 21. The hydrogel’s increased capacity to promote wound closure and re-epithelialization by keratinocytes was significant. The hydrogels exhibited excellent cytocompatibility and were non-cytotoxic in vitro. The expression of VEGF in the wound treated with the hydrogel was high at day 14, showing the correlation of HIF-1α with VEGF protein expression which indicates that HIF-1α promotes angiogenesis in deep tissue injuries [112].

### 4.2. Chitosan-Based In Situ Forming Hydrogels

In situ gels have been reported to be useful as wound dressings (Table 1). They display several advantages such as ease of administration, extended contact time of drug at the site (thereby improving drug bioavailability), reduced frequency of administration, improved patient compliance, etc. [113,114]. They are smart systems that represent a promising means of delivering drugs and they undergo sol–gel transition after administration. In situ gel formation occurs due to stimuli such as change in pH, temperature, etc. [115,116]. The application of in situ gels as wound dressing results from their capability to fit the irregular shape of the wound without causing any form of wrinkles [117].

Lin and co-workers formulated Histatin1 (Hst1)-modified thiolated chitosan-based hydrogels for wound dressing [118]. These hydrogels exhibited gelation time that ranged between 5 and 7 min at 37 °C in vitro while it was 8 min for the in situ injectable hydrogels in vivo. These results demonstrated that these hydrogels can quickly form in situ and can be utilized for skin defects repair in vivo. The drug release studies of the hydrogels in vitro at physiological conditions (pH 7.4 and 37 °C) using Rhodamine B (Rh B) and Rhodamine B–Hst1to stimulate the release mechanism of Hst1 peptide from the hydrogels showed that the release of Rh B increased within 72 h. The release of Rh B was higher than Rh B–Hst1 within 72 h. Furthermore, the Rh B–Hst1 release rate was decreased with an increase in the concentration of Rh B and Rh B–Hst1. The Hst1 was released in vivo via the degradation and the diffusion of chitosan. The wound healing studies of hydrogels in vivo at 0, 2, 5 and 7 days showed that the wound site contracted and the wound healing mechanism of Hst 1-loaded hydrogels were faster when compared to the free hydrogels [118].

Huang et al. formulated and evaluated antibacterial PEG diacrylate (DA)/chitosan hydrogels [119]. These hydrogels demonstrated in situ forming properties, adhesiveness, good mechanical strength, improved biocompatibility, and higher antibacterial activity. The PEG DA/chitosan hydrogels composed of 15 weight% of PEGDA and 2 weight% of chitosan displayed outstanding mechanical adhesiveness, sustained release of plasmid DNA and antibacterial peptides. Furthermore, the wound healing study in vivo displayed important acceleration in the wound healing mechanism in full-thickness skin defect mode by stimulating the angiogenesis and reducing the inflammation [119].

Lv et al. formulated in situ injectable hydrogels based on two biopolymers, carboxymethyl chitosan and alginate, for wound healing. The hydrogels containing 0.5% of chitosan oligosaccharide stimulated human umbilical cord mesenchymal stem cells proliferation and remarkably enhanced the wound healing mechanism in a mouse skin defect model. In addition, the microscopic wound analysis of the injectable hydrogels displayed an increase in the integrity and thickness of the epidermal tissue and increased the development of collagen fibers [120].

Song et al. designed a chitosan complex hydrogel for wound management. The rheological analysis revealed that chitosan-based hydrogels displayed a great anti-shear ability and broad linear viscoelastic region. The hydrogels exhibited moderate bactericidal efficacy against *S. aureus* and *E. coli*. The in vivo wound healing examination of chitosan-based hydrogels showed that the hydrogels significantly reduce wound size by 44% and 82.34% after 5th and 10th day, respectively [121].

Xu et al. designed in situ alginate-chitosan hydrogel for corneal wound healing via periodate-mediated sodium alginate oxidization. In vivo studies on alkali burn wounds in vivo showed improved epithelial reconstruction. Limbal stem cells were transplanted into the in situ hydrogel. The hydrogels provided favorable microenvironment for the cell differentiation and proliferation of the transplanted cells. The restoration of the integrity of the corneal after alkali burn was rapid. The in situ hydrogel displayed good biocompatibility, transparency, and biodegradability suitable for tissue regeneration [122].

Gholizadeh et al. developed chitosan-based in situ gels for the treatment of nasal wounds. Injury of nasal epithelium can lead to nose bleedings. The thermosensitive in situ gel was loaded with tranexamic acid. The formulation rapid liquid-to-gel phase change occurred in 5 min at the human nasal cavity temperature. Its liquid-to-gel capability retained the formulation in the anterior part of the nose with no lung deposition and also improved the drug bioavailability thereby prolonging the drug release on the wounded epithelium. The rate of wound closure was higher in cells treated with the in situ gel formulation after 1 h (6.89% ± 0.03) and 3 h (8.38% ± 0.03), when compared to treatment using the drug solution which was 1.16% ± 0.01 and 4.87% ± 0.01. The combination of factors such as the hemostatic properties of chitosan, the formulation of in situ gelation capability, sustained drug release profile and improved drug bioavailability at the wound site promoted the healing efficacy [123]. He et al. developed injectable hydrogel from catechol- and methacrylate-modified chitosan/gelatin. The formulation was effective against *P. aeruginosa* and *S. aureus*, killing more than 80% of these bacteria in a period of 2 h. It also displayed tissue adhesive that adhered to the skin at body temperature and it rapidly closed open wounds on the dorsum of a rat [124].

### 4.3. Cellulose-Based Hydrogels

There are also several hydrogels that are designed from cellulose and its derivatives for wound application (Table 1). Joorabloo formulated carboxymethyl cellulose-based hydrogels loaded with heparinized zinc oxide nanoparticle via a freeze–thaw method for application in wound dressing [125]. The average particle size of the loaded ZnO nanoparticles ranged between 16 and 36 nm. SEM images showed increased pore sizes after the addition of the nanoparticle with decreased pore density. The in vitro drug release profile at physiological conditions was an initial burst release of the heparinized nanoparticles from hydrogels followed by a sustained drug release. The mechanical properties of hydrogels increased with an increase in the amount of nanoparticles loaded into the hydrogel. The mechanical strength and Young’s modulus increased while the elongation at breakpoint decreased [125]. Cell viability study of hydrogels loaded with nanoparticles demonstrated non-toxicity on HDF and L-929 cells after 2 days. The wound healing assessment studies revealed good wound healing capability on the created artificial wound after 24 h. The antibacterial activity of the hydrogels using disc diffusion method showed significant bactericidal efficacy of over 70% for ZnO-loaded hydrogels against *S. aureus* and *E. coli* [125].

Gupta et al. prepared bacterial cellulose hydrogels encapsulated with curcumin for wound dressing application [126]. The water vapor transmission rate of the hydrogels was in the range of 2526.32–3137.68 g/m^2^/24 h suggesting that the hydrogels can provide a moist environment at the wound site. The in vitro drug release studies of curcumin from the hydrogels showed that 76.99 ± 4.46% of the loaded drug was released after 6 h followed by a slow and sustained drug release mechanism. The high bioavailability of the drug from the hydrogels at the wounded site was effective at controlling bacterial-associated infection. The curcumin-loaded hydrogels reduce oxidative stress at the wound site [126]. Fan and co-workers formulated and evaluated pH-sensitive dual drug-loaded cellulose-based hydrogels as potential wound dressings. In vitro degradation evaluation showed that the hydrogels degraded under slightly acidic conditions and the loaded drugs were released. The in vivo wound healing assessment of the hydrogels showed that during the wound healing process, the weight of the rats was maintained indicating that rats were in good health. Furthermore, these hydrogels promoted a high percentage of wound closure [127].

Erdagi et al. prepared gelatin–diosgenin–nanocellulose hydrogels [128]. Their morphology was interconnected with good porosity indicating that they are suitable for cell proliferation and adhesion. The gel yield of the hydrogels ranged between 83.67 ± 2.18% and 90.17 ± 3.51%. The hydrogels demonstrated good water uptake efficiency and reached equilibrium swelling within one day. The mechanical properties of the hydrogels showed compression modulus that ranged between 3.04 ± 0.15 kPa and 8.04 ± 0.31 kPa demonstrating good strength. These results from mechanical properties revealed these hydrogels as suitable scaffolds for application in wound healing. The in vitro antibacterial assessment of neomycin-loaded hydrogels showed higher bacterial inhibition against *S. aureus* when compared to *E. coli* [128]. Liu et al. formulated bacterial cellulose hydrogels with tailored crystallinity from *Enterobacter* sp. FY-07 by the controlled expression of colanic acid synthetic genes. These hydrogels exhibited significant water-holding capacity of 25,643% with good properties such as stability, tensile strength, purity, porosity, low immunogenicity, and biocompatibility. These properties are very important in dressings for wound management [129].

Shefa et al. prepared and evaluated oxidized cellulose nanofiber-PVA hydrogels incorporated with curcumin for wound healing acceleration [130]. The hydrogels displayed interconnected microscopic pores. The viscosity assessment showed that the viscosity of hydrogels was increased with increased in the polyvinylalcohol concentration and decreased by the addition of curcumin. The cell viability study of hydrogels using MTT assay showed over 100% cell viability on L929 cells while proliferation study in these cells displayed increased cell proliferation with the span of time (from day 1 to 7). The in vitro drug release profile of the hydrogels containing 7.5% and 10% polyvinylalcohol at pH 7.4 phosphate buffer saline solution revealed a sustained release of curcumin. In all of the hydrogels, curcumin release increased after 1 day of incubation. The wound healing analysis demonstrated the significant wound closure between 28.8 ± 1.3% and 29.9 ± 1.7% when compared to 8.3 ± 1.13% of the control [130]. Sulaeva et al. formulated bacterial cellulose-based hydrogels incorporated with alginate to improve their performance for wound management. These hydrogels demonstrated enhanced water retention properties. These scaffolds were moreover demonstrated to be biocompatible and useful for the treatment of bacterial colonized wounds [131].

Yang et al. formulated biocompatible and stable hydrogel composites that are based on dialdehyde carboxylmethyl cellulose cross-linked with collagen [132]. The high degree of crosslinking in these hydrogels was promoted by a high concentration of collagen because the ε-amino groups on the collagen is suitable for covalent crosslinking with aldehyde groups of dialdehyde carboxylmethyl cellulose. The high cross-linking capacity significantly contributed to the strong elastic behavior of the hydrogel. The stability of the hydrogel was enhanced by the formation of more covalent bonds, resulting in improved viscoelastic properties, resistance to enzymatic degradation and thermal stability. The cell viability studies on L929 fibroblasts using CCK-8 tests demonstrated that the dialdehyde carboxylmethyl cellulose hydrogels cross-linked with collagen did not promote any toxic cellular responses [132]. Fontes et al. designed and evaluated bacterial cellulose-based hydrogels for wound management. It was prepared from the combination of cellulose with either Calendula officinalis or Jacaranda caroba. The hydrogel provided a humid environment, promoted rapid tissue repair, reduced severe inflammation and did not damage granulation. The hydrogels showed faster re-epithelialization over a period of 7 days. The hydrogel was composed of an interconnected pores which promoted cell migration and rapid re-epithelialization. The hydrogels were suitable for dry wounds and their permeability to metabolites and decreased temperature of the wound bed can lead to reduced pain [133].

Liu and co-workers formulated cellulose nanocrystal reinforced nanocomposite hydrogels cross-linked with PVA with self-healing properties via a freeze–thaw cycle method for wound healing [134]. The SEM images of the hydrogels exhibited porous morphology and the pore size of the hydrogels decreased as the PVA amount increased. The mechanical properties analysis of the hydrogels displayed the compressive stress that ranged between 95 and 1056 kPa, with increased elasticity. Moreover, the Young’s modulus and tensile stress of the hydrogels increased from 0.52 to 9.9 MPa and 17 to 33 kPa, respectively, when the PVA amount increased. These results showed that PVA can improve the poor mechanical properties of cellulose. The self-healing efficiency of the hydrogels was improved by adjusting the quantity of amount of PVA and a PVA content of 3 g increased the healing efficiency of the hydrogels to 37.03% [134].

Jiji et al. prepared and evaluated thymol incorporated bacterial cellulose hydrogels for the management of burn wounds [135]. The thermal and chemical changes of the hydrogels were successfully evaluated. The hydrogel water vapor transmission rate decreased slightly after the loading of thymol into the polymer matrix because of thymol hydrophobic nature. The in vitro biocompatibility assessments using MTT assay showed that the hydrogels facilitated the growth of mouse 3T3 fibroblast cells, demonstrating low cytotoxicity, and increased cell viability. The in vitro antibacterial studies revealed that thymol incorporated hydrogels possessed an excellent zone of inhibition in both Gram-positive and Gram-negative bacteria strains, especially in the case of *S. aureus* and *K. pneumonia* when compared to the standard antibiotic, gentamycin. The in vivo wound healing studies of the hydrogels using female Wister rats with burn wound demonstrated that the wound was enlarged at the 5th day. On day 15, the burn wound area was significantly reduced by 55%. The wound treated with plain cellulose-based hydrogels and thymol-loaded cellulose hydrogels were reduced by 74.5% and 90.7%, respectively, on day 20 [135].

Sadegh and co-workers formulated carboxymethyl cellulose-human hair keratin hydrogels for the controlled release of clindamycin [136]. A higher keratin content in the hydrogel reduced the water vapor transmission rate of the hydrogels from 3200 ± 196 to 1921 ± 92 g/m^2^/day, which is suitable for wound management. The in vitro drug release mechanism at physiological conditions demonstrated an initial burst release of clindamycin from hydrogels during the first 4 h followed by a slow drug release profile. The total amount of released clindamycin after 7 days of incubating in phosphate buffer saline solution was 91.5 ± 3.1%, and it was decreased by the addition of keratin. This could be due to the hydrophilicity with increasing the keratin and lowering of water uptake value. These results suggest that the water uptake of the drug delivery system affects the released kinetics of hydrophilic drug (clindamycin). The in vitro antimicrobial activity of the hydrogel was 99.66% against *S. aureus* and this value was reduced by the addition of keratin because of the slow release of the antibiotic [136].

Deng et al. reported hydrogel prepared from fenugreek gum and cellulose. The composite hydrogel displayed a porous structure with good thermal stability and water absorption. The sustained release mechanism of the hydrogel was significant. The hydrogel also exhibited good biocompatibility and non-toxic properties. The hydrogels reduce blood loss and accelerated wound closure, with increased neovascularization and tissue repair [137]. Mohamad et al. reported bacterial cellulose/acrylic acid hydrogel loaded with human epidermal keratinocytes and human dermal fibroblasts for the treatment of burn wound. This in vivo study using athymic mice showed that the hydrogel loaded with cells accelerated burn wound healing. The % wound reduction on day 13 was (77.34 ± 6.21%) for the hydrogel when compared to the control which was (64.79 ± 6.84%). A high deposition of collagen was observed in the mice treated with hydrogel formulation which revealed their potential application for wound dressing [138]. Gupta et al. synthesized silver nanoparticles using curcumin:hydroxypropyl-β-cyclodextrin complex followed by loading into bacterial cellulose hydrogel. The hydrogels provided a moist environment with high cytocompatibility. They were effective against wound-infecting bacterial such as *Candida auris*, *Staphylococcus aureus* and *Pseudomonas aeruginosa* [139]. Koivuniemi et al. reported a single-center clinical trial evaluation of nanofibrillar cellulose wound dressing, (FibDex^®^ by UPM-Kymmene Corporation) and comparing it with a polylactide-based copolymer dressing. Twenty-four patients requiring skin grafting were enrolled for the study. The skin elasticity was improved with nanofibrillar cellulose wound dressing. The performance of the cellulose wound dressing was comparable to the polylactide when evaluated for the treatment of skin graft donor sites. It was self-detachable and did not require changing. It also reduced pain [140]. Deng et al. prepared hydrogel from cellulose and flaxseed gum for the treatment of bleeding wounds. The hydrogel exhibited a high thermal stability. Its swelling capability was high with a moisture uptake of 200%. The % drug adsorption was 7.27 ± 0.15 mg/g and was biocompatible; it promoted a hemostatic and wound healing process [141]. Khamrai et al. prepared a biocompatible gelatin–cellulose-based polyelectrolyte hydrogel patch with self-healing capability. It was prepared from bacterial cellulose, obtained from *Glucanoacetobacter xylinus* (MTCC7795) bacterial strain. Bacterial cellulose in the hydrogel enhanced the self-healing activity of the hydrogel. Incorporation of curcumin in the hydrogel enhanced their wound healing activity. The curcumin-loaded hydrogel inhibited the bacterial growth with a zone of inhibition value of 15 ± 0.5 mm and 19 ± 1.0 mm against *E. coli* and *S. aureus*, respectively. The presence of curcumin accelerated cell proliferation and the release of curcumin from the hydrogel was controlled [142].

Forero-Doria et al. prepared cellulose hydrogel which was conjugated with carbon nanotubes for high uptake of bioactive compounds (resveratrol, dexpanthenol, allantoin, and linezolid). The wound closure in vivo was accelerated. The release of linezolid was sustained. The hydrogel had the capability to protect the antibiotic from degradation. The hydrogel displayed good biocompatibility with L929 mouse connective tissue fibroblasts with cell viability of 95% [143]. Muchová et al. prepared poly(vinyl alcohol)-2,3-Dialdehyde cellulose hydrogel as wound dressings. The hydrogels displayed unique features such as high porosity, high drug-loading capacity, and good mechanical properties making them easy to handle with good adherence to the skin. They were also biocompatible and non-cytotoxic [144].

Wang et al. reported quaternized hydroxyethyl cellulose/mesocellular silica hydrogel via one-pot radical graft copolymerization for the treatment of bleeding wounds. The hydrogel loaded with mesocellular silica foam (9.82 *w*/*w*%) activated the blood coagulation factors. The quaternized hydroxyethyl cellulose reduced the plasma clotting time to 59% in vitro resulting in less blood loss. It also enhanced wound healing in a full-thickness skin defect model in vivo [145]. Li et al. prepared carboxymethyl cellulose/K-carrageenan/graphene oxide/konjac glucomannan hydrogel loaded with silver nanoparticles synthesized with green deoxidizer mango peel extracts. The hydrogel exhibited good antibacterial activity, biocompatibility and accelerated wound recuperating with the development of fibroblasts and rapid epithelialization [146].

## 5. Hydrogels Currently in Clinical Trials

Currently, some cellulose and chitosan are under selected phases of clinical trials or are marketed product as shown in Table 2. They were found to be effective for the management of different types of wounds.

## 6. Conclusions and Future Perspectives

The biopolymer hydrogels that are based on cellulose and chitosan demonstrated excellent features for the management of wounds. The hydrogels cross-linked with synthetic polymers displayed interesting mechanical properties that are useful in wound management, including good elasticity, flexibility, compressive stresses, Young’s modulus, and tensile stresses. The microbial infected wounds can delay the wound healing mechanism and increase wound exudate resulting in chronic wounds. These wound dressings possess antibacterial efficacy, especially those loaded with antibiotics or nanoparticles against several Gram-negative and Gram-positive bacteria strains that infect wounds. Furthermore, the hydrogels containing antimicrobial agents promoted the healing process by limiting the number of pathogens on the wound site. The use of biopolymer-based hydrogels in these wound dressings were characterized by increased porosity, high water uptake, non-immunogenic effects with sustained and controlled drug release. The aforementioned properties improved rapid re-epithelialization, granulation tissue development, and wound healing in vitro and in vivo. The combination of nanotechnology with medicine offers great opportunities to the currently available wound dressings. Nanoparticles are beneficial to improve the therapeutic efficacy of synthetic compounds. Nanoparticles can act on the cellular and subcellular events during the wound healing process. The incorporation of metallic nanoparticles and antibacterial agents into the chitosan or cellulose-based matrix promoted sustained antimicrobial activity without affecting the normal cell viability. The mechanism of action of metal-based nanoparticles is influenced by the concentration and is via the formation of free radicals. Despite the efficacy of nanoparticles, there is a pressing need to thoroughly investigate their toxicology and biocompatibility over long term application. Although biopolymer-based hydrogels present interesting features in the series of in vitro and in vivo studies reported for wound management, very few of them have reached clinical trials. Therefore, there is an increasing demand for the formulation of advanced wound dressing materials with enhanced properties and they must be preclinically tested to ensure safety. There is a great promise that many hydrogels that are based on cellulose and chitosan will enter the clinical trials and market in the near future.

## Figures and Tables

**Table 1:** Summary of hydrogels that are formulated from chitosan and cellulose.

Biopolymer Used for Hydrogel Formulation	Other Polymers Used	Loaded Bioactive Agents	Outcomes	References
Chitosan	PVP (Polyvinylpyrrolidone)	Silver sulfadiazine	Maximum swelling capacity, good antibacterial efficacy against Gram-negative *E. coli* strains with sustained and controlled drug release profile	[59]
Chitosan	Matrigel and polyacrylamide	**_**	Good mechanical properties and antibacterial activity	[60]
Chitosan	PVA Poly(vinyl alcohol)	ZnO nanoparticles	Good antibacterial efficacy	[61]
Chitosan	PEG Poly(ethylene glycol)	Ag nanoparticles	High porosity, good antimicrobial efficacy, and improved wound healing mechanism in diabetic wounds	[62]
Chitosan	**_**	SDF-1 α	Slow and controlled drug release kinetics	[63]
Carboxymethyl Chitosan	**_**	**_**	Excellent mechanical properties	[64]
Chitosan	**_**	**_**	Preventing hypertrophic scar development during the wound healing process	[65]
Chitosan	Alginate	Hesperidin	Good wound closure	[66]
Chitosan	Alginate	Alpha-tocopherol	Good wound contraction	[67]
N-carboxyethyl Chitosan	Pluronic F127	Moxifloxacin	pH-responsive drug release mechanism and good antimicrobial efficacy. Improved wound closure	[68]
Chitosan	_	Carotenoids	Accelerated wound healing process	[69]
Chitosan	**_**	**_**	Good hemostatic activity in a rat hemorrhaging and good antibacterial activity.	[70]
Chitosan	PVA and lignin	**_**	Accelerated wound healing with wound closure, good antibacterial activity, and good biocompatibility	[71]
Chitosan	**_**	Cefuroxime	Good biocompatibility on MG63 osteosarcoma and L929 fibroblast cell lines and excellent antibacterial efficacy	[72]
Chitosan	**_**	Gelatin and curcumin	Sustained and controlled drug release profiles	[73]
Chitosan	**_**	Human mesenchymal stem cells	Good cell viability, spreadability, and adhesive nature	[74]
Chitosan	**_**	Human mesenchymal stem cells	Improved full-thickness cutaneous wound healing	[75]
Chitosan	**_**	*P. granatum* peel extract	Good wound healing process	[76]
Chitosan	Pectin	Lidocaine hydrochloride	Sustained and controlled drug release	[77]
Chitosan	Polyurethane	Transplant bone marrow mesenchymal cells	Enhanced wound healing of diabetic wounds	[78]
Chitosan	**_**	Thymine oil cyclodextrin inclusion compound	Significantly decrease the number of bacteria	[79]
Chitosan	**_**	Silver nanoparticles	Non-cytotoxicity and high antibacterial activity against *E. coli* and *S. aureus*	[80]
Chitosan	**_**	Tetracycline hydrochloride	Excellent mechanical performance with superior antibacterial effects against *E. coli* and *S. aureus*	[81]
Carboxymethyl chitosan	Carbomer 940	*Bletilla striata*	Accelerated wound healing process and good blood compatibility	[82]
Chitosan	PVA	Silver nanoparticles	Excellent antibacterial activity against *P. aeruginosa* and *S. aureus*	[83]
Carboxymethyl chitosan	**_**	Melatonin	Increased wound closure with enhanced proliferation of the granulation tissue	[84]
Chitosan	**_**	Exosomes	Excellent wound healing effects	[85]
Chitosan	Lignin	_	Good biocompatibility	[86]
Chitosan	PVA and dextran	_	High cell proliferation	[87]
Chitosan	**_**	_	High water uptake with good antibacterial activity against *E. coli*.	[88]
Chitosan	**_**	Curcumin	Sustained drug release kinetics	[89]
N-carboxyethyl chitosan	Hyaluronic acid	Amoxicillin	High swelling capacity and wound healing process acceleration	[90]
Chitosan	**_**	Phenytoin	Good wound healing mechanism for diabetic and burnt wounds	[91]
Chitosan	PVA	S-nitroso-N-acetyl-DL-penicillamine	Good angiogenesis effects for wound healing process	[92]
Chitosan	PVA	_	Accelerated of wound healing process and re-epithelialization of burn wounds	[93]
Chitosan	PVA	Silver nanoparticles	Non-cytotoxicity with high antibacterial activity against *S.* *aureus* and *E. coli*	[94]
Chitosan	**_**	_	Effective bactericidal activity against *S. aureus* and *P. aeruginosa* with good wound healing effects	[95]
Chitosan	Gelatin	_	Good mechanical performance	[96]
Chitosan	Hyaluronic acid	_	Good biocompatibility with bactericidal efficacy against *S. aureus*	[97]
Chitosan	Xyloglucan	_	High porosity	[97]
Chitosan	**_**	_	Good biocompatibility	[98]
Chitosan	**_**	_	Good wound healing mechanism	[99]
Chitosan	**_**	Histatin1	High rate of wound healing process	[100]
Chitosan	**_**	Flavonoids	Increased lipid peroxidation at the injured tissue	[101]
Chitosan	**_**	Honey	High growth inhibition against various bacterial strains with accelerated wound healing process	[102]
Chitosan	**_**	Ibuprofen	Sustained drug release and good cohesiveness and adhesiveness	[103]
Hydroxybutyl chitosan	**_**	Human platelet lysate	Stimulated wound healing	[104]
Chitosan	**_**	_	Accelerated wound closure and high remodeling of extracellular matrix	[105]
Chitosan	**_**	Gentamicin	High antibacterial effects against *P. aeruginosa* and *S. aureus* with high cell viability and accelerated wound closure	[106]
Chitosan	**_**	Bemiparin	Accelerated the inflammation process and enhanced the re-epithelialization	[107]
Hydroxybutyl chitosan	**_**	_	Excellent biocompatibility and effective against *S. aureus* with good blood clotting capability	[108]
Chitosan	PVA	Cerium oxide nanoparticles	Bactericidal effective against MRSA with good biocompatibility	[109]
Chitosan	Cyclodextrin	_	Good hemostatic capability	[110]
Chitosan	Oxidized konjac glucomannan	Silver nanoparticles	Self-healing ability and good tissue adhesiveness	[111]
Chitosan	**_**	LL-37 peptide	High antibacterial efficacy against *S. aureus*	[112]
Chitosan	**_**	Histatin 1	Faster wound healing process	[118]
Chitosan	PEG diacrylate	Antibacterial peptide and plasmid DNA,	Important acceleration in wound healing mechanism on full-thickness skin defect model	[119]
Carboxymethyl chitosan	Alginate	Human umbilical cord mesenchymal stem cells	Remarkably speeded the wound healing mechanism in a mouse skin defect model	[120]
Chitosan	**_**	**_**	Significantly reduced wound size	[121]
Chitosan	Alginate	_	Rapid restoration of the integrity of the corneal after alkali burn	[122]
Chitosan		Tranexamic acid	The formulation was safe on human nasal epithelial cells with an efficient wound closure (six times faster than the control drug solution)	[123]
Cellulose	**_**	Curcumin	A potential antioxidant that can significantly reduce oxidative stress at the wound site	[124]
Cellulose	**_**	**_**	The high percentage of wound closure	[127]
Cellulose	Gelatin	Diosgenin	Good mechanical properties and higher bacterial inhibition	[128]
Cellulose	**_**	*Enterobacter* sp. FY-07	Significant water-holding capacity	[129]
Cellulose	PVA	Curcumin	100% cell viability, the sustained drug released, and significant wound closure	[130]
Cellulose	Alginate	**_**	Enhanced water retention properties	[131]
Carboxymethyl cellulose	Collagen	**_**	The better wound healing process	[132]
Cellulose	**_**	**_**	Enhanced wound healing process	[133]
Cellulose	PVA	**_**	Good mechanical properties and high wound healing efficiency	[134]
Cellulose	**_**	Thymol	Low cytotoxicity, increased cell viability, and reduced burn wound area	[135]
Carboxymethyl cellulose	**_**	Clindamycin	Good bactericidal activity	[136]
Cellulose	Fenugreek gum	_	Good biocompatibility and non-toxic	[137]
Cellulose	Acrylic acid	_	Accelerated burn wound healing	[138]
Cellulose	Hydroxypropyl-β-cyclodextrin	Silver nanoparticles and curcumin	Good antibacterial activity against various bacterial strains	[139]
cellulose	Polylactide	_	Self-detachable and did not require changing	[140]
Cellulose	Flaxseed gum	_	High swelling capacity with promoted hemostatic and wound healing process	[141]
Cellulose	Gelatin	Curcumin	Accelerated cell proliferation and the controlled release of curcumin from the hydrogels	[142]
Cellulose	_	Linezolid	Sustained drug release with accelerated wound closure and good cytocompatibility	[143]
Cellulose	PVA	_	High porosity, high drug-loading capacity, good mechanical properties	[144]
Hydroxyethyl cellulose	mesocellular silica	_	Enhanced wound healing	[145]
Carboxymethyl cellulose	K-carrageenan/graphene oxide/konjac glucomannan	Silver nanoparticles	Good antibacterial activity, biocompatible and accelerated wound recuperation	[146]

“_” means “No other polymer was used”.

**Table 2:** Hydrogels in clinical trials/marketed products.

Hydrogels	Polymer	Clinical Trial Outcomes/Marketed Products	References
Nanofibrillar cellulose wound dressing (FibDex^®^)	Cellulose	Efficient wound healing at skin graft donor sites, required no dressing changes, self-detaches after re-epithelialization, it did not degrade into tissue and it reduced pain.	[140]
Nanofibrillar cellulose wound dressing	Cellulose	Detachment of the wound dressing from epithelialized skin graft donor site is presented in occurred in average of 18 days.	[147]
Bacterial nanocellulose	Cellulose	Most of the patched skins did not show any symptoms of edema, vesicle and bullae. It was non-irritant and safe for the further evaluation.	[148]
Polyuhexanide-containing cellulose dressing	Cellulose	Clinical trials was carried out on patients with pressure ulcers infected with Methicillin-Resistant *Staphylococcus aureus.* There was a 100% eradication of the bacteria.	[149]
Nanoderm™ Ag	Cellulose	Displayed increased flexibility and sustained antimicrobial properties. Effective for the management of infected wounds.	[150]
Nanoskin^®^	Bacterial cellulose	It is 100% natural, non-allergenic, biocompatible, effective for the management of burns, surgical wounds, diabetic ulcer wounds, dermal abrasions, Skin grafting sites etc.	[150]
CelMat^®^	Bacterial cellulose	Useful for the treatment of burns, ulcers and chronic wounds. It promotes pain relief, excellent gases exchange, absorption and desorption of fluids.	[150]
EpiProtect^®^	Cellulose	Effective for the management of pediatric burn wounds after enzymatic debridement.	[151]
HemCon^™^	Chitosan	Good hemostasis activity and antibacterial barrier against some strains of bacteria.	[152]
KA01 chitosan wound dressing	Chitosan	It enhanced wound healing by facilitating wound re-epithelialization and reducing pain level. It was safe and effective for the management of chronic wounds.	[153]
Chitosan Mesh Membrane	Chitosan	The mesh chitosan membrane promoted good adherence, excellent hemostasis, re-epithelialization of the wound, reduced itching and pain.	[154]
Chitosan gel	Chitosan	The gel is displayed rapid hemostatic activity and prevents adhesion formation. It is suitable for the management of common complications of sinus surgery.	[155]
Axiostat^®^	Chitosan	It exhibits rapid hemostasis and is suitable for emergency trauma and accidents. It is easily removed from the wound site without leaving any residue.	[156]
ChitoRhino	Chitosan gel	Good hemostasis activity and effective for wound healing after endoscopic sinus surgery.	[157]
ChitoHeal	Chitosan	Accelerates the rate of healing, scar reduction, biocompatible, effective for burns, cuts, scratches and diabetic foot ulcers	[158]
KytoCel	Chitosan fibers	It is a highly absorbent dressing suitable for the management of moderate and heavily exuding wounds.	[159]
PosiSep^®^	Chitosan fibers	A nasal dressing, easy to use, and display rapid expansion upon hydration for minimal bleeding procedures.	[160]
ExcelArrest^®^ XT	Chitosan-based patch	It accelerates the clotting process to control bleeding from the skin.	[161]
ChitoClot Pad	Chitosan	It undergoes gelation after absorbing blood and prevents exudation of absorbed blood.	[162]
XSTAT	Chitosan	It is used to treat gunshot wounds.	[163]
Chitoderm^®^ plus	Chitosan	Good absorbent properties.	[157]
ChitoClear^®^	Chitosan	A good hemostatic agent resulting in its capability to attract negatively charged red blood cells.	[164]
Celox™	Chitosan	Rapid hemostatic property and reduces blood loss.	[157]
